# Systems Medicine 2.0: Potential Benefits of Combining Electronic Health Care Records With Systems Science Models

**DOI:** 10.2196/jmir.3082

**Published:** 2015-03-23

**Authors:** Taavi Tillmann, Alexander R Gibson, Gregory Scott, Oliver Harrison, Anna Dominiczak, Phil Hanlon

**Affiliations:** ^1^Department of Epidemiology & Public HealthUniversity College LondonLondonUnited Kingdom; ^2^College of Medical, Veterinary and Life SciencesUniversity of GlasgowUnited Kingdom; ^3^Division of Brain SciencesImperial College LondonUnited Kingdom; ^4^Healthways InternationalLondonUnited Kingdom; ^5^Johns Hopkins Bloomberg School of Public HealthBaltimore, MDUnited States

**Keywords:** gene-environment interaction, systems theory, electronic health records, epidemiology, online social networks, crowd-sourcing, Web 2.0

## Abstract

**Background:**

The global burden of disease is increasingly dominated by non-communicable diseases.These diseases are less amenable to curative and preventative interventions than communicable disease. This presents a challenge to medical practice and medical research, both of which are experiencing diminishing returns from increasing investment.

**Objective:**

Our aim was to (1) review how medical knowledge is generated, and its limitations, (2) assess the potential for emerging technologies and ideas to improve medical research, and (3) suggest solutions and recommendations to increase medical research efficiency on non-communicable diseases.

**Methods:**

We undertook an unsystematic review of peer-reviewed literature and technology websites.

**Results:**

Our review generated the following conclusions and recommendations. (1) Medical knowledge continues to be generated in a reductionist paradigm. This oversimplifies our models of disease, rendering them ineffective to sufficiently understand the complex nature of non-communicable diseases. (2) Some of these failings may be overcome by adopting a “Systems Medicine” paradigm, where the human body is modeled as a complex adaptive system. That is, a system with multiple components and levels interacting in complex ways, wherein disease emerges from slow changes to the system set-up. Pursuing systems medicine research will require larger datasets. (3) Increased data sharing between researchers, patients, and clinicians could provide this unmet need for data. The recent emergence of electronic health care records (EHR) could potentially facilitate this in real-time and at a global level. (4) Efforts should continue to aggregate anonymous EHR data into large interoperable data silos and release this to researchers. However, international collaboration, data linkage, and obtaining additional information from patients will remain challenging. (5) Efforts should also continue towards “Medicine 2.0”. Patients should be given access to their personal EHR data. Subsequently, online communities can give researchers the opportunity to ask patients for direct access to the patient’s EHR data and request additional study-specific information. However, selection bias towards patients who use Web 2.0 technology may be difficult to overcome.

**Conclusions:**

Systems medicine, when combined with large-scale data sharing, has the potential to raise our understanding of non-communicable diseases, foster personalized medicine, and make substantial progress towards halting, curing, and preventing non-communicable diseases. Large-scale data amalgamation remains a core challenge and needs to be supported. A synthesis of “Medicine 2.0” and “Systems Science” concepts into “Systems Medicine 2.0” could take decades to materialize but holds much promise.

## Current Limitations in the Study and Management of Chronic Disease

Medical science has brought clear and dramatic improvements to health over the past 150 years. This has included effective cures and preventions as well as important public health measures. As a consequence of many of these advancements, the leading causes of death and disability have shifted from infectious diseases to more complex non-communicable diseases. In the past 50 years, expenditure on health research and health care has increased dramatically to meet the new challenges that treating and preventing these multifaceted diseases presents. However, combating chronic disease has proven significantly more difficult and costly than infectious disease, with it becoming increasingly difficult to continue raising life expectancy and healthy life expectancy [1]. Many spheres of academia and clinical practice have shifted away from an intent to cure, to an attempt to slow down pathological processes and prevent complications. This is in part due to the inability of science to determine which individuals will suffer the most from any given risk factor, or who will benefit most from specific interventions.

Current medical science is largely conducted using the reductionist paradigm, which assumes that complex entities are best understood by breaking them down into smaller, simpler components. Detailed analysis of the weaknesses of this assumption has been done elsewhere [2], but principally, reductionism limits our ability to understand how multiple variables interact with one another to create emergent effects.

The reductionist approach does offer a useful first step for the understanding of a complex system because it helps identify key components. However, a strong and enduring emphasis on the reductionist approach risks over-simplification (focusing only on a handful of major factors with the biggest effect, while the sum of minor factors may be considerable) and generalization (assuming that a common cause-effect relationship applies equally in all cases). Moreover, an excessive focus on a limited number of pathways may impede our ability to understand both the behavior of the system as a whole, as well as system variability between individuals. Our etiological models of seemingly disparate chronic diseases include a striking number of common pathways. For example, alterations of the tumor necrosis factor alpha (TNF-alpha) gene have been implicated in 88 clinically distinct diseases [3]. However, it remains unclear under what circumstances increased TNF-alpha levels cause an individual to develop rheumatoid arthritis, atherosclerosis, or a septic cytokine cascade. It is assumed that many such pathways interact to produce disease outcomes. Rarely can we adequately describe why a disease develops in an individual, their prognosis, the effect of risk factor modification on an individual, or the likelihood of family being affected by a similar disease.

The type of drugs dispensed to patients further reflects our limited understanding of the true cause of disease. While some drugs can reverse the original disease process to provide a near cure (eg, antibiotics, chemotherapy), many more provide only temporary relief to the current physiological imbalance and fail to cure (eg, thyroxin, antidepressants, anticonvulsants, steroids, diuretics). Others still simply dampen the body’s capacities to exacerbate the condition in a symptomatic or palliative [4] fashion (eg, beta blockers, warfarin) with significant side effects. Another weakness in our management of chronic diseases stems from the way research studies and clinical guidelines consider each disease in isolation, while in reality most patients have at least one comorbidity. This leaves patients with an expanding burden of treatment, polypharmacy, increased side effects, unintended drug interactions, and reduced adherence.

This pattern of diminishing returns necessitates a reevaluation of the approach that medical science has taken in the study and management of chronic diseases. As chronic diseases are substantially more complex than infectious diseases, additional approaches are probably required to overcome the limitations of reductionism. In this essay, we aim to (1) document medical science’s first steps in moving away from reductionism towards more complex models, (2) assess the potential benefit of introducing Systems Science into medical science and evaluate the relative strengths and weaknesses of various technologies in facilitating this (such as Medicine 2.0), and (3) present our suggestions of how to best increase medical research efficiency. To achieve this, we combine an unsystematic review of peer-reviewed literature, with a review of two websites known for the dissemination of novel technologies and ideas (Wired, TED). We then try to bring together these disparate lines of thought, across a range of disciplines and industries, into a subjective but hopefully thought provoking synthesis.

## Supplementing the Reductionist Paradigm

Should the prevalent theoretical model, framework, or paradigm find it increasingly difficult to account for experimental data, alternatives to the prevalent model should be considered [5]. This can be done by replacing the old model or by adding supplements to the existing model [6]. We argue that three such supplements have recently been added to the core of reductionism. First, select chemotherapy agents were found to work particularly well for subtypes of breast cancers and leukemia, which corresponded to genetic subtypes of the disease. This reveals that diseases may look similar on the outside but can function very differently on the inside. As our knowledge of this etiome [3] (the precise etiological pathways by which genetic and environmental agents cause a disease) grows, it becomes useful to name each diagnostic subcategory with appropriate subdivisions. The term “intermediate pathophenotype” [7] has been suggested to capture these subdivisions. Linguistically, this denotes a focus on end-state pathology, the likely object of interest for pathologists and systems biologists. Clinicians, epidemiologists, and biomedical scientists are, however, more interested in upstream etiology and the narration of an individual’s past and likely future. Perhaps adopting the term “etiphenotype” instead would make Systems Medicine more accessible to clinicians (or alternatively, letting the term “etignosis” complement “diagnosis”).

The second supplement to reductionism came from the observation that most patients have more than one disease at any given time and that some diseases tend to cluster together. For example, people with diabetes have a greater risk of developing certain other diseases, such as stroke. Recent evidence has identified numerous early commonalities, such as “the metabolic syndrome” or “diabesity”, but our scientific language and modes of thought struggle to describe the interconnectedness of these tightly intertwined etiological processes [8]. Reductionism is ill-suited to deal with the question of comorbidity, prompting a search for alternative models [9]. Third, in many cases it appears better to treat complex diseases with a cocktail of drugs administered simultaneously. This has been applied in the treatment of infections (eg, tuberculosis and human immunodeficiency virus), as well as for chronic conditions (eg, acute coronary syndrome, exacerbations of chronic obstructive pulmonary disease) [10]. Advances in such complex, combined interventions have prompted the Medical Research Council to issue guidance on how they should be monitored and evaluated [11]. We believe that this will give only modest improvements, as intervention development will still remain somewhat blind and ignorant of the intricacies of etiological processes.

## Modifying the Reductionist Paradigm With Network Medicine

**Figure 1:**
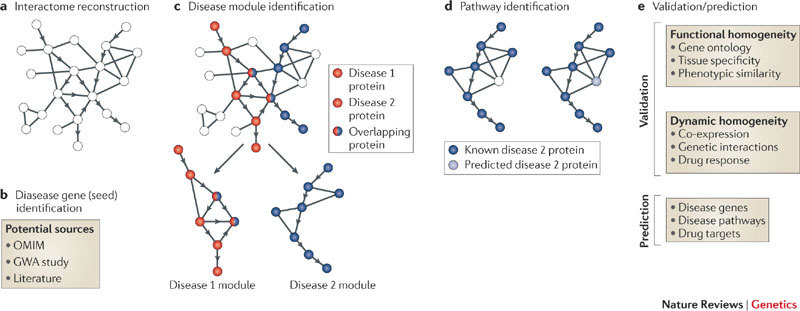
The methodological steps by which network medicine identifies disease modules and predicts novel protein targets for intervention.

A transitionary model of medical research is emerging that attempts to reconcile some of the discrepancies outlined above. There is much hope that network medicine could be better suited to understand the basic biological processes that culminate in health and disease [12,13]. This framework suggests that complex diseases are the emergent result of perturbations in multiple genes that are interconnected to one another to create a disease module (Figure 1). A hypothetical study of diabetes could begin by mapping the system of proteins that are responsible for healthy function like glucose regulation (Figure 1, panel a), also known as the interactome of proteins. Next, existing literature is used to identify a candidate gene or protein critical in etiology (Figure 1, panel b). This protein is mapped onto the interactome to identify a smaller set of proteins that directly influence the candidate protein (Figure 1, panel c), to identify a suspected diabetes disease module (colored red). Concurrent mapping of comorbid diseases (such as stroke in blue) can identify structural reasons for comorbidity as well as common premorbid states (such as the metabolic syndrome, marked by the red-blue proteins). Models like this can account for the effectiveness of combined interventions (Figure 1, panel d, dark blue proteins) and identify novel drug targets (Figure 1, panel d, light blue). Such models have been used to identify novel genes across a range of cancers [14-16]. It has also allowed drugs to be re-positioned for other diseases, such as using the anti-ulcer drug cimetidine in the treatment of lung cancer [17].

We believe that the network medicine approach can achieve half the paradigm shift required toward systems medicine. However, such models remain incomplete because of the following weaknesses [18]. (1) When considering disease, network medicine looks only at snapshots of end-stage illness. The model cannot be applied to longitudinal data to describe the gradual shift that takes place when healthy states slowly transform into diseased states. This weakness stems from the model’s implicit assumption that disease processes (such as glucose dysregulation) are merely broken flipsides of healthy processes (glucose regulation). The cascade of events that caused the shift from healthy state to diseased state are irrelevant and not investigated [19]. (2) Network medicine focuses exclusively on the intracellular level of proteins and genes. Researchers interested in higher order risk factors (such as how weight gain is influenced by childhood nutrition, family upbringing, socioeconomic status, or the physical environment of green spaces and fast food outlets) struggle to utilize network medicine models [20]. (3) Simplistic causal modeling, wherein diseased genes and proteins are thought to exert their effects uniformly across a range of interindividual and interenvironmental variation. Thus network medicine struggles to model how genetic risk interacts with environmental risk to create disease. We feel that these three weaknesses will keep our models of disease largely incomplete, thus perpetuating many of the shortcomings outlined in our introduction.

## Replacing the Reductionist Paradigm With Systems Medicine

**Figure 2:**
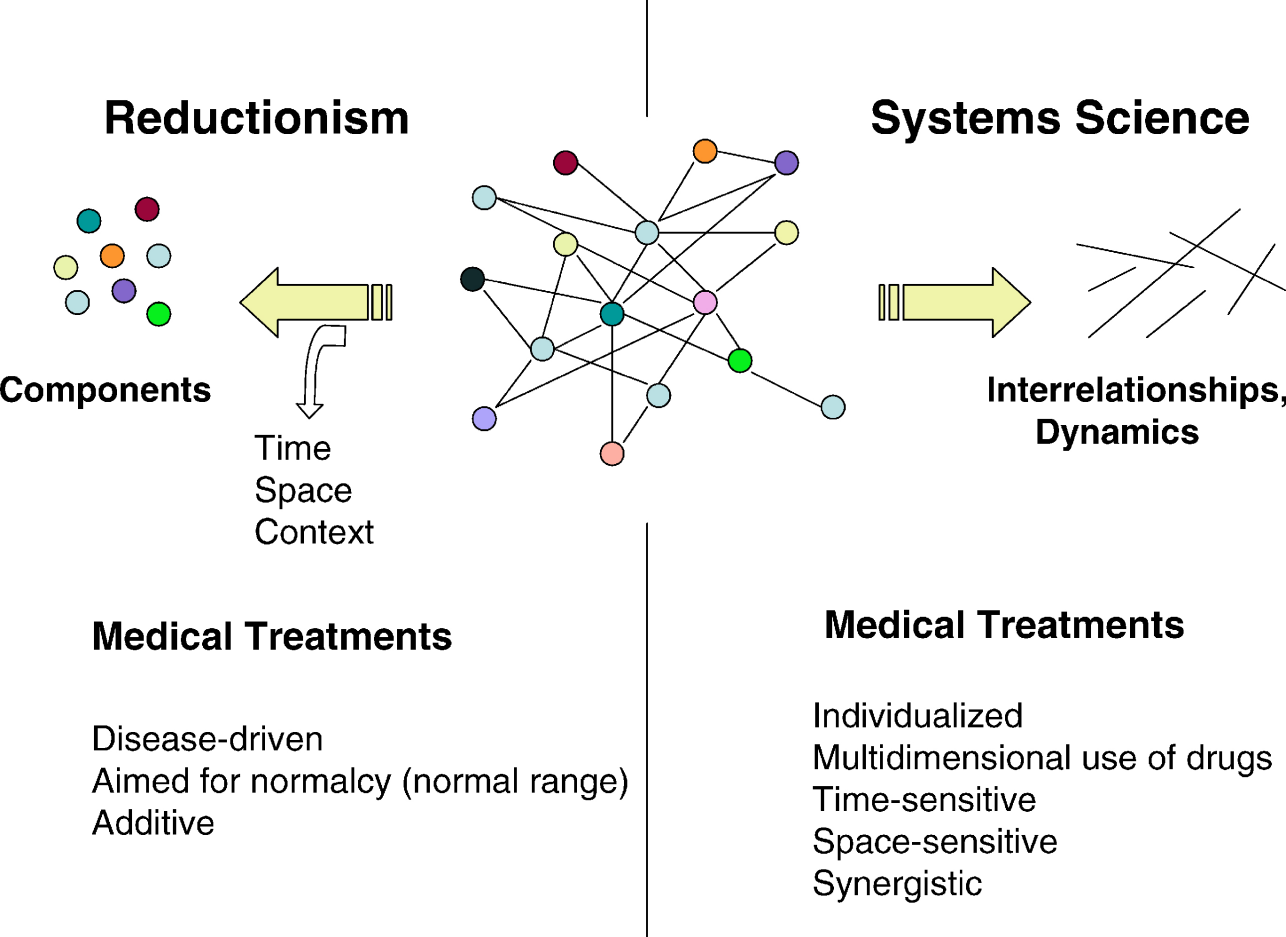
Schematic illustration of the core differences between reductionism and systems science, when analyzing the properties of a system.

It helps to begin by clarifying our core terms. *A System* can be defined as a set of components that are related to one another in a meaningful way (centerpiece, Figure 2). *Systems Science* is the formal study of systems [21]. *Systems Thinking* describes modes of thought that focus on the connectedness and interrelationships of components, rather than focusing on the components themselves. *Systems Theory* is a set of theories that try to derive generalizable organizing principles that apply to all systems. For example, complex systems can maintain robust performance during external perturbations, thanks to system-wide properties such as *modularity* and *built-in redundancy* (ie, having two kidneys), features that are seen in biological and non-biological systems alike [19]. *Systems Biology* is the study of how biological functions emerge from the interactions between the components of living systems and how these emergent properties in turn influence the behavior of lower-level components [22]. The field developed quickly during the past decade, due to technological advances in generating large amounts of high-throughput data very quickly, along with the interdisciplinary ability to compute, model, and make sense of this data.

To date, little of the above paradigms have been incorporated into medical research. In part, this may be due to difficulties in amalgamating large-scale clinical datasets. However, as such technical barriers begin to fall away, increasing attribution can be placed on lack of knowledge about Systems Science among the medical research community. Indeed, those looking for a working definition of Systems Medicine may be baffled by the diversity of opinions out there [19,23,24]. Historically, Systems Medicine has been defined as the clinical application of Systems Biology approaches to medicine, where traditional model-driven experiments are informed by data-driven models in an iterative manner [25]. We see Systems Medicine as the long-term objective of a wider paradigm shift in medical science, at the end of which a range of different models and approaches will coexist under the Systems Medicine umbrella. All of these models will be substantially more complex than the models used in Reductionism or Network Medicine. We suggest that Systems Medicine models should include two or more of the following organizing principles of the human body: non-linearity, multi-agency, multi-levelness, or adaptivity [26].

*Non-linearity* means that the independent variables interact with one another and modify each other’s effects on the dependent variable. This makes the dependent variable exhibit emergent properties that can be understood only when *all* of the independent variables are assessed concurrently. Such interactions have been documented between behavioral risk factors, between two genes [27], between transcribed mRNA and regulatory microRNA [28], between single nucleotide polymorphisms (SNP) and expression quantitative trait loci [29], as well as between transcription factors [30]. Perhaps most important of all are gene-environment interactions, which have begun to emerge for obesity [31], coronary artery disease [32], asthma [33], colorectal cancer [34], depression [35], eczema [36], Alzheimer’s disease [37], and multiple sclerosis [38] to name a few. Models of systems medicine need to be built and empirical data assembled so that the detection of interactive effects and non-linear dynamics is facilitated. Contrast this with the stance taken in most introductory epidemiology and medical statistics courses, where interactive effects are seen as “nuisance” phenomena and students are discouraged against opening such cans of worms because of seemingly unmanageable type 1 errors. Regrettably, the study of interaction phenomena remains a niche field. This stifles progress at understanding any non-linear mechanisms of the human body.

Non-linearity can be present in relatively simple systems [39]. Consider the example of a system where smoking and drinking alcohol leads to increased stroke risk, through the upregulation of a hypothetical inflammatory cytokine called “smokdrink”. These two risk factors can interact with one another and produce non-linear responses that are quite complex in nature (such as a dramatic increase in smokdrink, but only if you drink more than 3 units a day *and* have accumulated more than 20 years of smoking damage). Nonetheless, our model remains simply non-linear (Multimedia Appendix 1, frame 1). Next, we can expand the model to account for how stroke is further influenced by another non-linear system, namely the cholesterol system (frame 2). If we view smokdrink and cholesterol as our two agents of stroke, then we have built a multi-agent model (frame 3). This concept is useful to bear in mind as the outcome of complex systems is rarely determined by one agent, but rather is the interaction between multiple agents (Multimedia Appendix 1, red arrows at the top of frame 3). Many of these agents remain directly unmeasurable, but their parameters can be estimated. For example, even before we discover the inflammatory cytokine smokdrink, we can calculate that 20 cigarettes + 3 pints a day has a similar effect on stroke, as does 10 cigarettes + 5 pints a day. Constructing intermediary agents *in silico* (such as smokdrink and cholesterol) facilitates our conceptual and mathematical understanding of the nature of dynamic disease processes. Such constructs could also include the inflammatome [40], the Metabolic Syndrome scale, the Hypothalamus-Pituitary-Axis dysregulation scale, or the allostatic load scale [41].

Multi-level models account for how people often form natural groups, which in turn influence individual behavior and disease outcomes. A grouping variable, such as “living in a deprived neighborhood or not”, can exert its effect on the disease outcome directly just like any other risk factor (Multimedia Appendix 1, frame 4, dotted blue line), or indirectly by modifying the effect of a lower order risk factor (such as drinking alcohol; Multimedia Appendix 1, red dotted lines). Although it may be tempting to enter “living in a deprived neighborhood” into the model alongside the other variables, this violates certain statistical assumptions. Accordingly, models known interchangeably as nested, hierarchical, or multiscale models, are useful to take account of the multi-levelness of such real-life phenomena.

Finally, all the models presented so far compare states of full health against states of full illness. The sequence of events over which full health deteriorates towards full illness remains unknown. To understand this process, we must build dynamic models of the human body. The core principle here is *adaptivity*, wherein after a certain stimulus, the system modifies its own response pattern in anticipation of similar stimuli in the future. It is through processes such as down-regulation, long-term potentiation, habituation, and synaptic learning, that the body modifies its definitions of “an ideal state” or “an ideal response”. Thus the rules that explain the behavior of homeostatic systems are nested in larger systems, called homeodynamic systems [41]. Imagine an individual whose homeostatic system is trying to keep its blood glucose level around 6, and Body Mass Index (BMI) around 22 (also known as an attractor zone). The system tolerates and shows resilience in the face of a range of perturbations in the short-term (such as skipping a meal, met by glycogenolysis), medium-term (entering Ramadan, met by lipolysis), or permanent (increased energy demands, met by increasing appetite). Whatever happens, the system will try to return to the initial attractor zone. This is easy to do if the person’s state meanders only slightly in the immediate phase space around the central attractor zone, also known as the system’s *basin* (eg, glucose 4-8, BMI 20-24). However, if the person’s state moves far away enough from the basin (eg, glucose 2, BMI 10), the integrity of the system is at stake and death from starvation may follow. In other diseases, the attractor zone may shift to BMI 26 (diagnosed as overweight) or to glucose 12 (diagnosed with diabetes) over a few decades. Other diseases still may show less gradual but more sudden shifts with bifurcation into a far away attractor zone (as seen in an acute epileptic seizure [42], many other acute presentations, but also in agent-based models of community interventions [43]). Other diseases can show cyclical flip-flop behavior between two attractor zones (such as is seen in bipolar disorder [44]). Dynamical Systems Theory focuses on understanding how and why these attractor zones change over time. Chaos Theory describes a type of system change that is particularly sensitive to initial conditions (such as seen in the Barker and Hygiene hypotheses). Similar models have been used to understand cell fates [45], endothelial function [46], cytokine function [47], heart rate variability [48], atrial fibrillation [49], septic shock [50], and multiple organ dysfunction syndrome [51]. Understanding these dynamic processes may lead to targeted interventions of preventative [52] or curative nature. For example, animal models suggest that infection with helmliths can reprogram autoimmune disease states towards healthier ones in colitis [53], gastric atrophy [54], multiple sclerosis [55], and diabetes [56].

## Analytical Approaches to Systems Medicine

Conducting systems medicine research will require numerous changes to how medical scientists and epidemiologists conduct their daily work. Such changes have been poorly addressed in the existing literature. Fundamentally, an entire new approach to data collection and analyses is required. If most risk factors interact with one another to create small interactive effects, many of which are nested in intricate multi-levels of hierarchy, then the detection of such intricacies will require large numbers of patients with large numbers of variables [57].

Once assembled, such high-fidelity datasets could be explored for “hot spots”, or loci of association, between risk factor data (including behavioral, environmental, and sociological risks), molecular data (including genomic, trascriptomic, and proteomic data), and clinical outcomes, in order to identify and treat etiphenotypical subgroups. Some of this exploration will take the narrow shape of testing a priori hypotheses. Other exploration will use high-throughput techniques to scan more broadly and identify novel hypotheses for subsequent scrutiny [58,59]. This may be facilitated by machine learning algorithms [60]. For example, the world’s most advanced supercomputer, IBM Watson, has recently been given access to data on over a million cancer patients, as well as the emerging oncological literature. It is hoped that Watson will support and enhance clinical decision making in real time [61]. This may shatter the traditional view of humans generating hypotheses on which computers calculate the test statistic, as computers like Watson may also become indispensible in hypothesis generation. Our vision of Systems Medicine becomes impossible without seamless collaboration with mathematicians, modelers, and data scientists, so that the right modeling tools can be used and combined to appropriately balance sufficient complexity with practical utility.

## A Roadmap to Systems Medicine

Although multiple challenges impede the integration of systems approaches within medical research, we prioritize five that deserve attention first. (1) Existing models that view the human body as complex adaptive systems are limited and need specialist development. Our review of the literature suggests that while textbooks exist on modeling for Systems Science and Systems Biology, little consensus exists on modeling for Systems Medicine. (2) While large-scale research projects (such as the Human Genome Project, Wellcome Trust Case Control Consortium, Virtual Physiological Human, ECell, Cancer Genome Atlas, Human Brain Project) are developing at a promising rate, the majority of research continues to be conducted in an almost proprietary-like mindset of non-sharing, with individual teams collecting and holding data in small silos. This makes it difficult to create the large datasets required for systems analysis. (3) While some organizations (like IBM, Sage Bionetworks, and Farr Institute) can foster multidisciplinary teams between medical researchers and information specialists, this collaboration gap remains large in most areas of public research. (4) Although research funders are beginning to recognize and invest in long-term and larger research endeavors, particularly in database curation, the vast majority of funds continue to be spent on low-complexity projects with short-term deliverables and seemingly low risk. Many such projects are underpowered in breadth, depth, and complexity to sufficiently address the problems they seek to address, with sometimes negligible subsequent improvement in our knowledge base. (5) The obstacles above will yield with sufficient energy and leadership; however, progress at each front continues to be slow due to the prevalent reductionist culture in the medical research community and general risk aversion to engage with new technology (particularly those that may alter existing power relations). Therefore, greater discussion, awareness, and education of differences between systems medicine and reductionism are required across the board, in order to promote and facilitate interest and activity in this important transition.

## Sharing Data

The medical research community itself is a complex adaptive system. Shifting this toward a more efficient, systems-science configuration will require time and effort to be applied at multiple levels. We believe that the second obstacle mentioned above, creating large datasets, is most pressing so we discuss this in detail. There are two possible non-competing solutions: (1) sharing data between researchers or (2) using data from electronic health records (EHR) for research purposes. Although data sharing appears to be increasing, most research data are not shared or recycled outside the original project team. A range of factors discourage data sharing. Project-specific data are often collected using context-specific priorities, definitions, and measurement tools that are rarely compatible with other peer-researchers, let alone researchers looking at the picture from a higher or lower level of order [62]. Data access requests are often cumbersome and slow, and some researchers may be wary of giving up their competitive edge [63]. Ultimately, even if all research groups pool their data successfully, a rich trove of clinical process and outcome data will continue to be held by health care providers who are not involved in research.

Health care data are never assembled for research purposes, which substantially hampers its transferability. For example, patients with borderline disease states are often “upcoded” into more severe disease states, as this brings financial rewards to the health care provider. Some EHR data may always be absent or of insufficient quality to be useful in medical research (eg, tracking the daily improvement of inpatients with cellulitis or fluid overload, which is difficult to quantify). However, other EHR data lend themselves very well to scientific analyses, particularly as drug prescription data are of exceptional quality. Furthermore, as health care is carried out on real patients, studying the real-life effects of drugs is preferable to unrepresentative clinical trials, particularly in understanding how one drug can accidentally influence two disparate diseases [64,65]. Future work could clarify which health care data are transferrable to medical research, and develop some tools to facilitate this. For example, should some severe diagnoses, when seen in conjunction with other hospital laboratory parameters, be downcoded back to a more truthful level, to correct for financial upcoding in EHR? The integration of health care and research data will create its own technical, logistical, and legal challenges, and at present we cannot tell if this will be worthwhile. If attempted, we suggest that groups of patients could be subdivided empirically based on their differential risk factor profiles, disease trajectories, or molecular data [66], and these subgroups subsequently explored in more detail for evidence of etiphenotypical subgroups.

## Unlocking Health Care Data

There is growing support for the collation of EHRs for secondary research purposes [67]. Transmission of data is typically thought to proceed with researchers asking health care providers for access to anonymized data [68]. Added value can be gained by integrating data from multiple providers [69]. Overall, this approach offers substantial productivity gains. However, this benefit must be considered against the drawback of reduced data accuracy.

As an alternative, we suggest an approach where researchers ask patients directly for their data, who in turn have to ask hospitals for their data. The advantage here is that routine medical data could be augmented by study-specific scientific tests (such as genotyping). The disadvantage is that samples will not be representative of the wider patient group, but biased towards those comfortable with sharing their personal data with scientists over the Internet, a concept that probably frightens most patients today. It also requires EHRs to be personally accessible and personally controlled—a feature that is still under development [70], but with some proof of concept from private settings [71].

## Online Communities

Web 2.0 technology is creating a social Web, where users create content, share useful information with interested peers, and moderate each other’s activities (eg, Facebook, eBay, ReseacherGate) [72]. Web 2.0 has been combined with the power of crowdsourcing (the force behind projects like Wikipedia) to foster Science 2.0 (also known as Open Science or Cyberscience 2.0) [73]. Here, online platforms facilitate large-scale data sharing between researchers who can merge or re-analyze each other’s data (eg, HapMap project, Sage Bionetworks). Some of these projects (eg, GalaxyZoo) are also enlisting the power of citizens in the scientific endeavor. A seminal article by Eysenbach in 2008 [74] detailed how the application of these Web 2.0 technologies to personally controlled health care records will create “Medicine 2.0”. This will be a world where the collaboration gap between researcher, clinician, and patient will narrow. Online patient communities such as PatientsLikeMe are not only useful to facilitate peer transfer of knowledge [75] but also in scientifically testing new drug applications [76].

**Figure 3:**
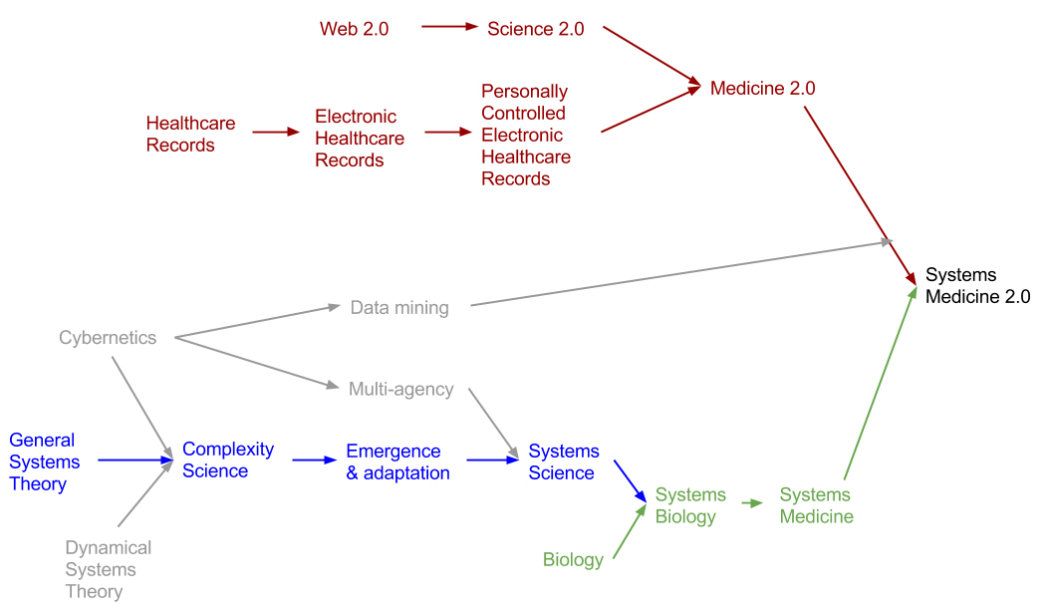
The historical origins of “Systems Science” and “Medicine 2.0”, and the potential for their combination into “Systems Medicine 2.0”.

To the best of our knowledge, a major gap in the current literature is the fact that systems medicine researchers have never suggested that their vision could be achieved quicker using Medicine 2.0 tools. Similarly, although Medicine 2.0 enthusiasts have begun to associate their ideas to Big Data, Web Science, Health Web Science, and the Semantic Web [77], none have spoken of the potential for Medicine 2.0 to further our understanding of the human body as a complex adaptive system. We urge these two important communities to begin collaboration on what we suggest could be called “Systems Medicine 2.0” (Figure 3)*.* Understandably, few patients will trust their most sensitive secrets with an amorphous, faceless research community. An online community with detailed researcher profiles, faces, and credentials (eg, ReseacherGate) could be augmented by allowing participants and researchers to interact with one another. Reliable feedback systems (such as those seen in Amazon and eBay) will be central to creating sufficient trust. To date, most sites have focused on fostering researcher-researcher collaboration [78,79], or patient-patient collaboration. The next step would be to foster bi- or tri-directional links (also to clinicians), with the added challenge of increased knowledge asymmetries.

Another challenge for Systems Medicine 2.0 is how health care data has very poor interoperability, both nationally and internationally. We welcome the work of the Joint Initiative on SDO Global Health Informatics Standardization to facilitate the rapid adoption of common health informatics standards. A related challenge is how various Systems Medicine research teams will initially create their own datasets, analytical programs, and models. Reporting these via traditional journals will hamper progress. Datasets, programs, and models should be shared, scrutinized, and developed, perhaps by making use of the efficiency tools perfected by the Open Source movement [80]. Open access [81], open standards, open source software [82], and open competitions [83] can foster faster innovation and drive efficiency. Ideally, the entire Systems Medicine community could see themselves as working on the same project, akin to the global communities who brought us Linux, Mozilla Firefox, Wikipedia, HUGO, and Systems Biology.

To start the discussion of how the research community can move towards Systems Medicine, we have compiled a list of suggestions (Textbox 1). This is neither exhaustive nor comprehensive and is designed merely to stimulate discussion, development, and implementation of Systems Medicine.

**Initial suggestions for the advancement of systems medicine.**

Governments and key research funders:

- training systems scientists, big data engineers, and medical researchers
- incentivizing and normalizing multidisciplinary collaboration for systems medicine
- incentivizing and normalizing the sharing of laboratory data as well as health care data
- earmarking research funds for systems medicine
- national leadership on systems medicine strategy and implementation

Legislation for the Web 2.0 era:

- EHRs to be recognized as patient property
- EHR systems must facilitate the simple and free export/import of data by abiding to global standards and/or open standards
- anonymized research on EHRs is permitted and automatically facilitated, unless patients opt out
- define if and how data can be accessed for commercial purposes and how any proceeds are divided

Scientific community:

- opinion leaders to promote the benefits of Systems Science approaches in medicine
- disease-specific expert bodies (eg, colleges) to facilitate common standards of data collection in their fields
- early Systems Medicine 2.0 academics to focus their papers at one flagship journal (eg, *Journal of Medical Internet Research*)

Software developers:

- develop an online community that links together systems scientists and modelers, big data engineers, medical researchers, and subsequently patients

International co-ordination:

- international leadership and co-ordination (eg, World Health Organization) to promote all of the above
- an online data-sharing directory listing all large datasets pertaining to Systems Medicine

## Conclusion

The prevalent paradigm in medical science is reductionism, whose limitations have become increasingly apparent, resulting in diminishing returns. This paradigm has been supplemented and modified with the framework of network medicine. If further developed with Systems Science approaches, this has the potential to evolve into fully fledged Systems Medicine paradigm. This neatly complements advances in Internet-powered medicine (Medicine 2.0), but as yet the two fields have yet to take advantage of each other’s nascent existence. We hope that our paper can bridge this conceptual gap and advance mutual interest and collaboration between these two, to foster Systems Medicine 2.0. This could lead to significant advances in the prevention and treatment of non-communicable diseases.

## Multimedia Appendix 1

A PDF illustrating various aspects of a Systems Medicine model of disease: non-linearity (frame 1), multi-agency (frame 3), multilevelness (frame 4).

## Abbreviations

BMI: Body Mass Index
EHR: Electronic Health Record
