# Supplementary material for: Systems Medicine 2.0: Potential Benefits of Combining Electronic Health Care Records With Systems Science Models
Source: J Med Internet Res. 2015 Mar 23;17(3):e64. doi: 10.2196/jmir.3082 (PMC4387294; doi:10.2196/jmir.3082)
Supplement: Supplementary file 1 [file jmir_v17i3e64_app1.pdf]

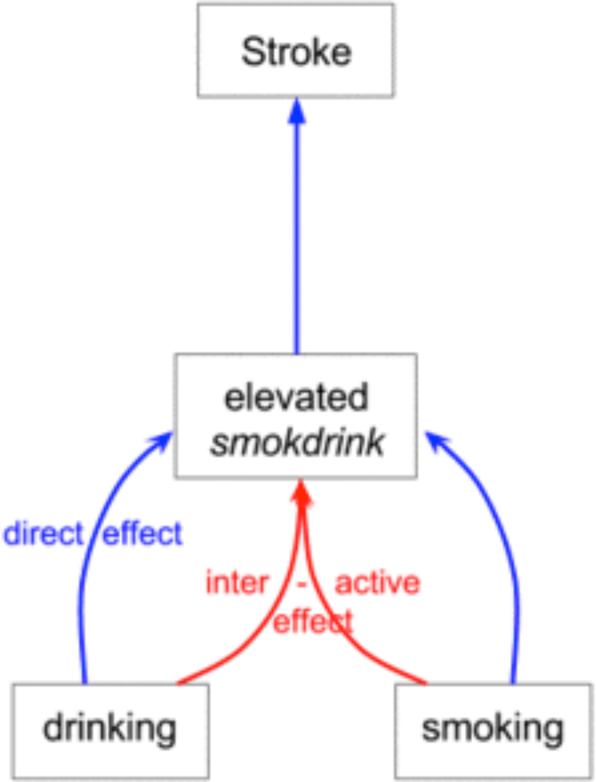


*Frame 1 - A simple, nonlinear system*

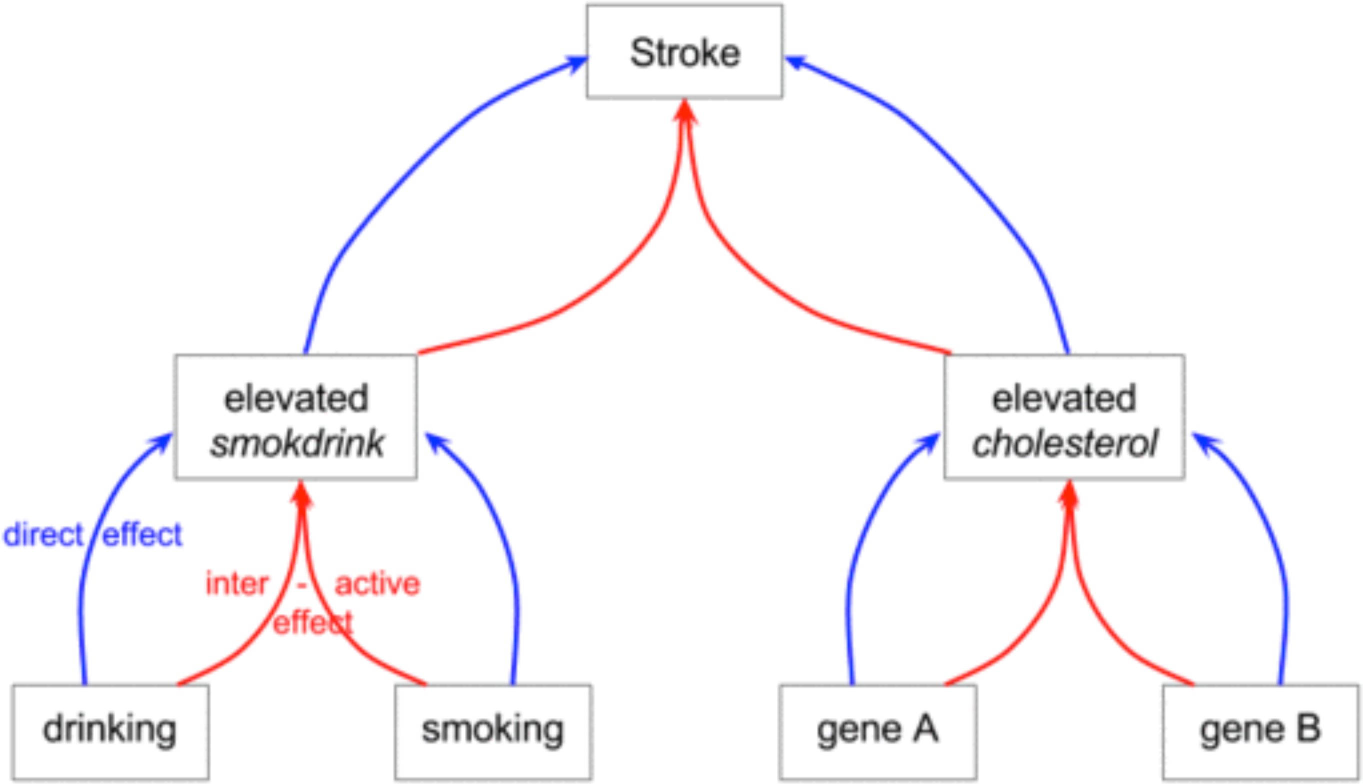


*Frame 2 - increasing complexity*

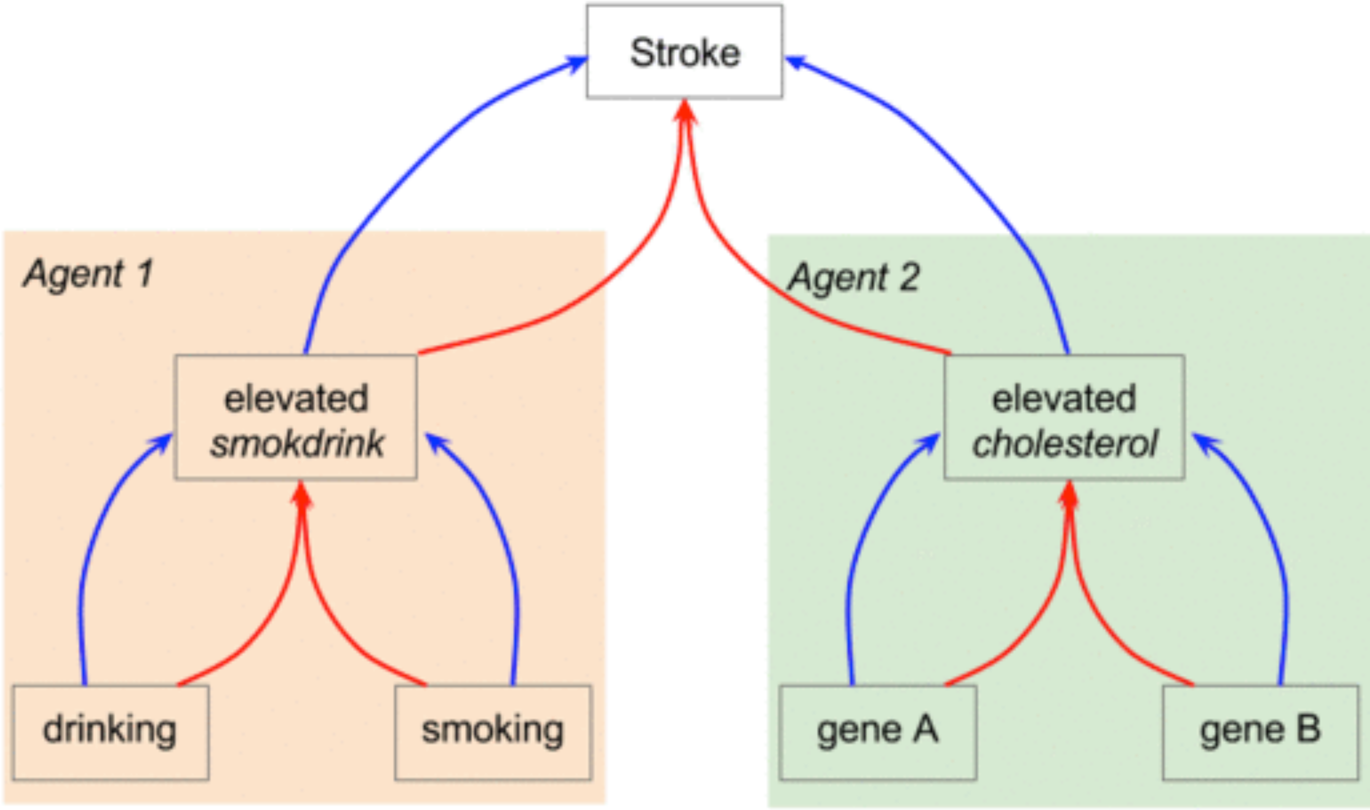


Frame 3 - a multiagent system

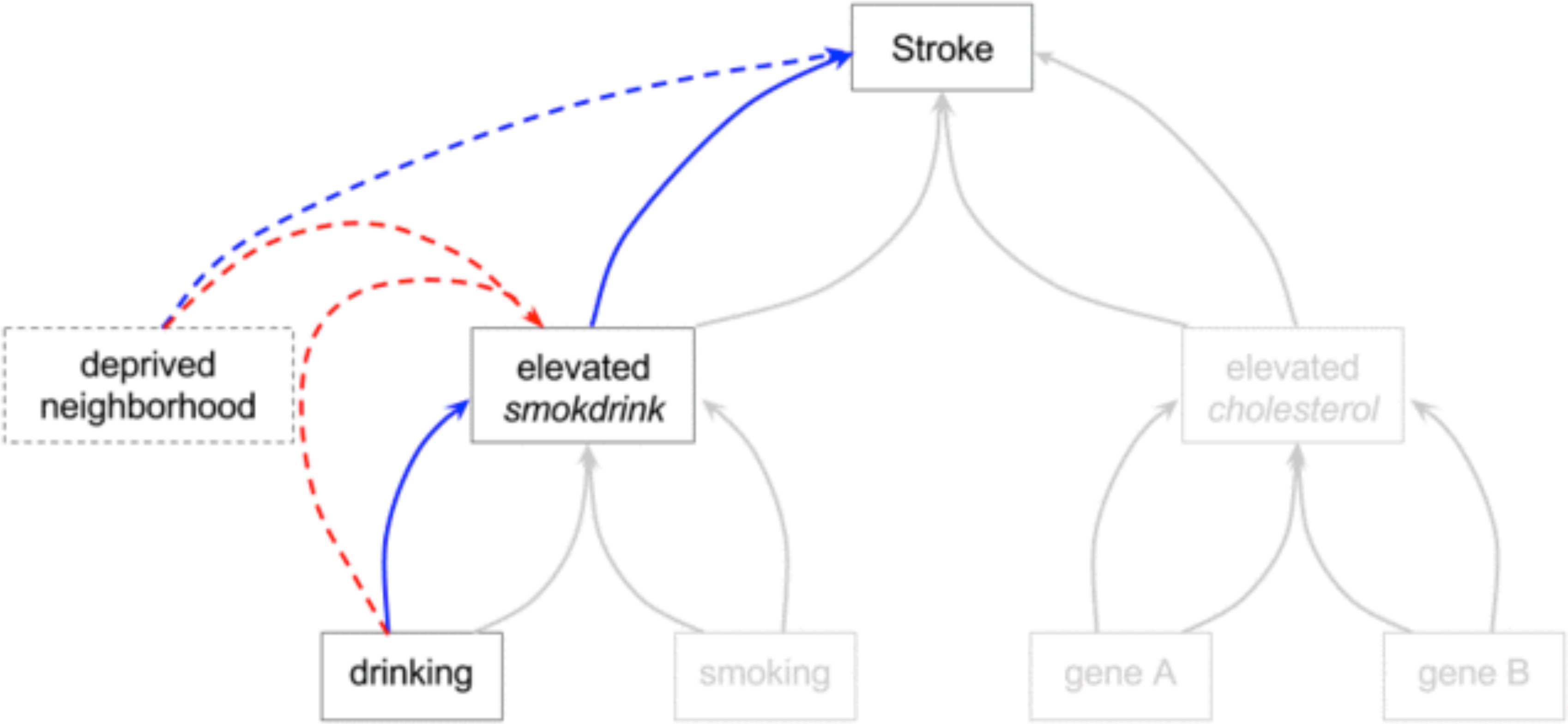


*Frame 4 - a multilevel system*
